# Deep Neural Networks Based on Sp7 Protein Sequence Prediction in Peri-Implant Bone Formation

**DOI:** 10.1155/ijod/7583275

**Published:** 2025-04-07

**Authors:** Pradeep Kumar Yadalam, Carlos M. Ardila

**Affiliations:** ^1^Department of Periodontics, Saveetha Dental College, SIMATS, Saveetha University, Chennai, Tamil Nadu, India; ^2^Department of Basic Sciences, Biomedical Stomatology Research Group, Faculty of Dentistry, University of Antioquia, Medellín, Colombia

**Keywords:** bone regeneration, deep learning, osteoblast differentiation, peri-implantitis, protein sequence analysis

## Abstract

**Objective:** Peri-implant bone regeneration is crucial for dental implant success, particularly in managing peri-implantitis, which causes inflammation and bone loss. SP7 (Osterix) is vital for osteoblast differentiation and bone matrix formation. Advances in deep neural networks (DNNs) offer new ways to analyze protein sequences, potentially improving our understanding of SP7's role in bone formation. This study aims to develop and utilize DNNs to predict the SP7 protein sequence and understand its role in peri-implant bone formation.

**Materials:** and Methods: Sequences were retrieved from UniProt IDs Q8TDD2 and Q9V3Z2 using the UniProt dataset. The sequences were Sp7 fasta sequences. These sequences were located, and their quality was assessed. We built an architecture that can handle a wide range of input sequences using a DNN technique, with computing needs based on the length of the input sequences.

**Results:** Protein sequences were analyzed using a DNN architecture with ADAM optimizer over 50 epochs, achieving a sensitivity of 0.89 and a specificity of 0.82. The receiver operating characteristic (ROC) curve demonstrated high true-positive rates and low false-positive rates, indicating robust model performance. Precision-recall analysis underscored the model's effectiveness in handling imbalanced data, with significant area under the curve (AUC-PR). Epoch plots highlighted consistent model accuracy throughout training, confirming its reliability for protein sequence analysis.

**Conclusion:** The DNN employed with ADAM optimizer demonstrated robust performance in analyzing protein sequences, achieving an accuracy of 0.85 and high sensitivity and specificity. The ROC curve highlighted the model's effectiveness in distinguishing true positives from false positives, which is essential for reliable protein classification. These findings suggest that the developed model is promising for enhancing predictive capabilities in computational biology and biomedical research, particularly in protein function prediction and therapeutic development applications.

## 1. Introduction

Peri-implant bone regeneration restores bone tissue around dental implants damaged by peri-implantitis or other bone loss factors [1]. This process is crucial for controlling peri-implant illnesses and ensuring dental implant success and stability. Inflammation in the soft and hard tissues around dental implants is called peri-implantitis [2]. Symptoms include inflammation, bleeding on probing, bone loss, and implant failure. Dental implant health depends on peri-implantitis prevention and management. Regenerating bone tissue around dental implants afflicted by peri-implantitis [3] or other bone loss factors is known as peri-implant bone regeneration. Peri-implantitis therapy has limited success and seeks to minimize bleeding on probing, enhance probing depth, and fill a vertical bone defect using novel techniques. Preventing bone loss and ensuring dental implant success require regular monitoring, care, and oral hygiene [4].

Bone-forming proteins [2, 3] establish a complicated milieu for osteoblast function. Collagen type I scaffolds protein and mineral deposits, bone sialoprotein (BSP), osteocalcin, and osteopontin attract calcium and other minerals to the bone matrix [5, 6]. bone morphogenetic proteins (BMPs) promote osteoblast growth and differentiation. After that, osteoblasts create collagen type I and other proteins. Once the bone matrix is created, osteoblasts become osteocytes [7].

SP7 protein, or Osterix (Osx) [8], is a transcription factor essential for osteoblastogenesis and bone production in mice and humans. The Sp subfamily of zinc-finger transcription factors, which includes SP7, is extensively conserved in bone-forming vertebrates. SP7 [9], a sequence-specific DNA-binding protein, regulates osteoblast differentiation and bone formation by targeting gene transcription. Bone development and osteoblast differentiation require Osx, the transcription factor SP7. Osx strongly controls osteoblast differentiation and function. It promotes MSC growth into pre- and mature osteoblasts via transcription. Bone matrix formation and mineralization require osteocalcin, BSP, and collagen type I genes. Osx works with Runx2 [10], another essential transcription factor (Cbfa1). Runx2 is needed for MSC commitment to the osteoblast lineage, and Osx supports osteoblast development later in differentiation.

According to research, Osx is essential for differentiating mesenchymal progenitor cells into bone-forming osteoblasts. It clusters with osteoblast-associated extracellular matrix (ECM) genes, such as BSP and new proteoglycans [8]. The concentration and duration of bone morphogenetic protein-6 (BMP6) influence gene expression. In the presence of BMP6, Osx overexpression increases ECM gene expression and promotes osteoblast mineralization, while Osx knockdown inhibits differentiation. The SP7 gene is located on human chromosome 12 (12q13.13) and mouse chromosome 15. Alternative splicing produces a full-length 431-residue isoform and a shorter 413-residue isoform [11]. The SP7 gene consists of three exons. Genome-wide association studies have strongly linked the human SP7 locus to bone mineral density and bone mass [12].

One previous study examined the roles of the zinc finger-containing transcription factor Osx/Specificity protein-7 (Sp7) in osteoblast differentiation. The results show that Sp7 inhibits the proliferation of immature osteoblasts, induces osteoblast maturation and Col1a1 expression, and is necessary for osteocytes to develop adequate survival processes, thereby avoiding cortical porosity[13]. This insight is vital for focusing on Sp7 when designing osteoporosis and peri-implantitis treatments. These studies showed that Sp7 is a good target for bone formation and that SP7 sequences, also known as Osx, are vital DNA-binding sites that serve as transcription factors that regulate osteoblast differentiation and bone formation. They primarily bind to AT-rich regions with proteins like Dlx factors to activate bone-specific genes. Research on rat bone marrow mesenchymal stem cells (BMSCs) showed that circRNA422 significantly promotes osteogenic differentiation, evidenced by increased alkaline phosphatase, SP7 transcription factor, and LRP5 levels. Silencing circRNA422 reversed these effects, indicating its importance in this process. Additionally, SP7-binding sites on the LRP5 promoter suggest a direct regulatory relationship [14].

Predicting SP7 sequences is crucial for understanding bone development, osteoporosis, regenerative medicine, drug development, evolutionary studies, and biomarker identification. SP7 plays a critical role in osteoblast differentiation and function, and its sequences can provide insights into bone development and related disorders. Aberrant expression or mutations in the SP7 gene can lead to conditions like osteoporosis and osteogenesis imperfecta. Knowledge of SP7 sequences can aid in developing regenerative therapies for bone repair and regeneration, potentially contributing to stem cell therapy. Targeting SP7 or its pathways in drug design could lead to new therapeutic modalities for treating bone-related diseases or enhancing bone healing. Predicting SP7 sequences can be done using bioinformatics tools, gene annotation, sequence alignment, machine learning approaches, experimental validation, and gene expression studies. Deep neural networks (DNNs) [15] analyze intricate patterns within extensive datasets and find applications in image recognition, natural language processing, and machine translation. Each layer of a DNN processes data uniquely, enabling the model to forecast outcomes for new data after training [16, 17]. Recent studies have developed DNN models for protein peptide sequence classification [17–19]. These models train a single network to recognize different features and generate representations of distinct protein activities using layers, allowing the same low-level features for different high-level classifications [20]. Such an approach leverages the computational power of DNNs to uncover intricate patterns in protein sequences that traditional methods might overlook, enhancing the accuracy and reliability of protein function predictions [21–23].

Identifying key sequence features of SP7 critical for osteoblast differentiation and bone matrix formation can contribute to improved therapeutic strategies for peri-implant bone regeneration. This study aims to develop and utilize DNNs to predict the SP7 protein sequence and understand its role in peri-implant bone formation.

## 2. Materials and Methods

### 2.1. Dataset Preparation

SP7 FASTA sequences were downloaded from UniProt IDs Q8TDD2 and Q9V3Z2 [18]. These sequences were identified, checked for quality, and subjected to preprocessing steps like FASTA sequences for sequence prediction using DNNs. To ensure data quality and model compatibility, several preprocessing steps are necessary. These include a sequence quality check, which ensures the downloaded sequences are complete and error-free, length filtering, and redundancy removal. Sequence cleaning involves trimming nonstandard characters, removing gaps, and standardizing sequences.

### 2.2. DeepBIO

DeepBIO [19] is a comprehensive web service for academics to develop deep-learning architectures to address various biological questions. DeepBIO can train and evaluate deep-learning models, compare and optimize models, and perform visualization analysis on biological sequencing data. For sequence and base functional site annotation, DeepBIO offers well-trained deep-learning architectures for over 20 tasks, complete with interpretations, graphical visualizations, and conservation motif analysis to verify site reliability. These sequence-based datasets were divided into training and test sets using DeepBIO. Each dataset was randomly partitioned into 1,000 training sets and 200 testing sets to optimize hyperparameters and evaluate performance.

### 2.3. Model Architecture

We used a DNN approach [21] to construct an architecture capable of receiving a wide range of input sequences, with computing requirements based on input sequence length. This model can parallelize training and prediction computations across the sequence. We aimed to develop networks to extract functional information from raw amino acid sequences through multi-label training on full-length proteins.

### 2.4. Model Parameters

**Table:** 

cuda: True
seed: 43
num_workers: 4
num_class: 2
kmer: 3
mode: train-test
type: prot
Model: DNN
Datatype: user provides
interval_log: 10
interval_valid: 1
interval_test: 1
epoch: 50
optimizer: Adam
loss_func: CE
batch_size: 8
LR: 0.0001
reg: 0.0025
gamma: 2
alpha: 0.25
max_len: 207
dim_embedding: 32
minimode: modelCompare
if_use_FL: 0
if_data_aug: 0
if_data_enh: 0

## 3. Results

**Figure 1:**
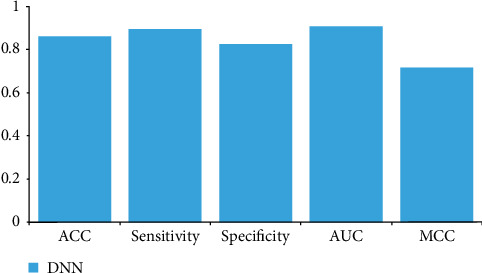
Accuracy and sensitivity of protein sequences.

FASTA protein sequences were subjected to deep neural architecture to identify hidden features and weights, and backpropagation algorithms were applied to fine-tune the model with ADAM optimizer and epoch–50 iterations.  Figure 1 shows the accuracy and sensitivity of protein sequences.

Table 1 shows the accuracy of the model. The proportion of positive cases correctly identified by the model is called sensitivity or the true-positive rate (TPR). It is calculated as follows: Sensitivity = TP/(TP + FN) = 0.89. Specificity, or the true-negative rate (TNR), is the percentage of negative cases the model correctly identifies. It is calculated as follows: Specificity = TN/(TN + FP) = 0.82. Sensitivity and specificity are complementary metrics, meaning that improving one often results in a decrease in the other.

**Figure 2:**
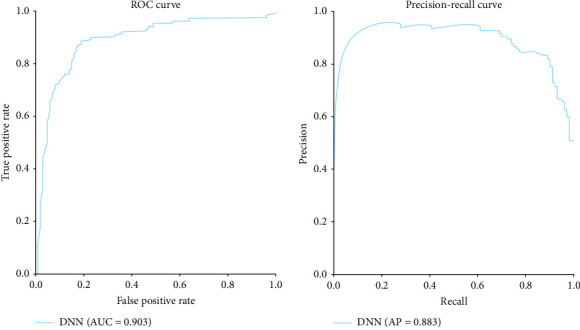
Model performance.

Figure 2 demonstrates that the model's performance shows improved accuracy with reduced false positives. The model's ROC curve shows it is a strong classifier, exhibiting a low false- positive rate (FPR) and a high TPR. It has an AUC of 0.903 and a precision–recall curve of 0.883, indicating effective performance in handling imbalanced datasets.

### 3.1. Receiver Operating Characteristic (ROC) Curve

The ROC curve illustrates the trade-off between a model's TPR (sensitivity) and FPR (1–specificity) across various classification thresholds. An ROC curve closer to the plot's upper left corner indicates a higher TPR and a lower FPR.

### 3.2. Precision–Recall Curve

A precision–recall curve (PRC) displays the trade-off between precision and recall for binary classifiers at different probability thresholds. Precision is the fraction of true-positive predictions among all positive predictions, while recall is the fraction of true positives correctly identified by the model. This curve reveals the model's performance, particularly with imbalanced classes. Classifier performance is often summarized by the area under thePRC (AUC-PRC), where a greater AUC-PR indicates better model performance.

**Figure 3:**
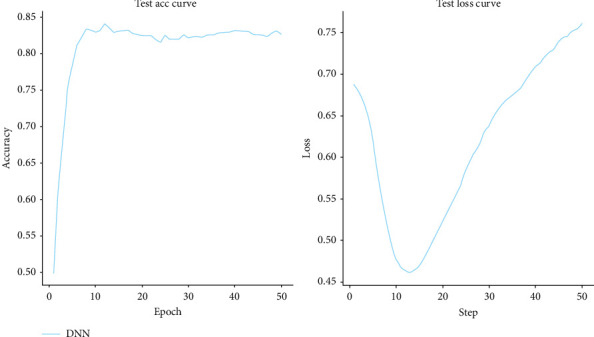
Epoch plot for iterations.

Figure 3 displays the Epoch plot illustrating iterations. An epoch plot is a graphical representation of a machine-learning model's loss and accuracy during training. It is a valuable tool for identifying overfitting and other issues with the model. The x-axis of an epoch plot represents the number of epochs or iterations used to train the model, while the y-axis represents either the model's loss or accuracy. Loss indicates how effectively the model predicts the correct output for a given input, while accuracy denotes the percentage of correct predictions made. This plot indicates high accuracy.

**Figure 4:**
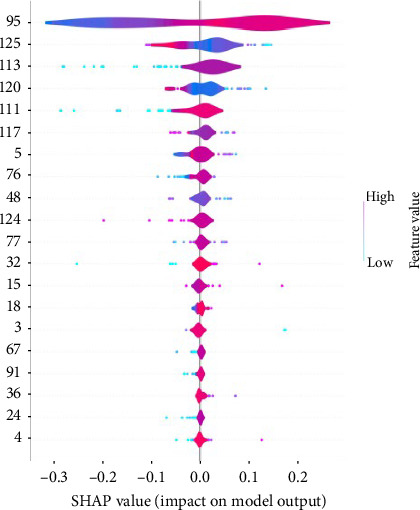
SHAP measure of model prediction and importance.

The SHAP plot is a visualization tool that uses horizontal and vertical axes, violin plots, color coding, and interpretation to analyze and validate features' contributions to a model's output. Feature 95 has the most significant impact on the model's output, with higher values leading to increased predictions, indicated by positive SHAP values. Feature 125 also substantially influences predictions, following a similar trend where high values enhance outcomes. In contrast, lower features, such as 4 and 24, exhibit minimal influence, as their SHAP values are primarily around zero (Figure 4).

## 4. Discussion

Sp7 [12], a downstream target in the Wnt signaling pathway, plays critical roles in cellular development, proliferation, and differentiation. It is particularly pivotal in guiding mesenchymal stem cells toward becoming osteoblasts, which are crucial for bone formation. Sp7 regulates the expression of key genes involved in bone development, such as BMP and collagen. Additionally, Sp7 influences the development of odontoblasts, which produce dentin, by controlling the expression of genes like DSPP and DMP1 [22–24].

Mutations in the SP7 gene are associated with several bone diseases [25, 26]. For instance, recessive osteogenesis imperfecta, characterized by bone fragility and delayed motor milestones, can result from frameshift mutations in SP7 [27, 28]. Dysregulation of Sp7 is also linked to osteoporosis, a condition characterized by low bone mass density [29].

The human transcription factor Sp7, also known asOsx, is encoded by the SP7 gene and, along with Runx2 and Dlx5, is crucial for the differentiation of mesenchymal precursor cells into osteoblasts and osteocytes. Understanding sequence variations and predicting novel protein structures using artificial intelligence and DNN are essential for advancing our knowledge of bone formation through Sp7 sequencing analysis [30].

Deep learning holds great promise for revolutionizing biomedical tasks, although challenges persist. While there have been incremental advancements, the interpretability of model predictions and the availability of labeled data remain significant hurdles. For instance, DeepFRI, a graph convolutional network, performs better in protein function prediction by integrating sequence features from protein language models and protein structures, including homology models [31 ].

Various methods, such as Rosetta [32], EVmutation [32, 33], and DeepSequence [34], have been utilized to predict sequence performance using established physics-based and unsupervised learning approaches. These methods facilitate studying sequence–function relationships within protein networks, capturing nonlinear interactions and sharing parameters across sequence sites.

In our research, we employed deep learning architectures to address the clinical challenge of peri-implant bone formation. Specifically, we applied deep learning sequence prediction to Sp7 protein sequences (Table 1), achieving an accuracy of 85% (Figures 1–4) in DNNs with strong sensitivity and specificity (89% and 82%, respectively) on test data.

While our study achieved promising results in predicting SP7 protein sequences for peri-implant bone formation, several limitations and future avenues for research should be considered. First, the interpretability of deep learning models remains a challenge, necessitating further investigation into how predictions are made and how these insights translate into biological understanding. Additionally, the availability of large, well-annotated datasets is essential for improving the robustness and generalizability of predictive models in biological contexts. Future studies should focus on integrating multi-omics data and leveraging advancements in structural biology to validate predicted sequences experimentally. Moreover, exploring ensemble learning techniques could enhance prediction accuracy and reliability. Addressing these aspects will be critical for advancing the utility of deep learning in understanding SP7's role in bone formation and its implications for clinical therapies.

## 5. Conclusions

Utilizing a DNN architecture with ADAM optimizer achieved a high accuracy of 0.85 in protein sequence analysis, demonstrating the effectiveness of deep learning in this domain. Solid sensitivity metrics (0.89) and specificity (0.82) were obtained, which are crucial for accurately evaluating computational biology and medicine predictive models. The ROC curve showed high true-positive rates and low FPRs, highlighting the model's robustness in protein sequence classification. Precision-recall analysis indicated the model's capability to handle imbalanced data, evidenced by a significant area under the curve. Epoch plots demonstrated consistent model accuracy throughout training, validating its reliability for protein sequence analysis in various biological and clinical applications. Therefore, the DNN model demonstrated high accuracy in predicting Sp7 protein sequences, facilitating the identification of novel proteins crucial for peri-implant bone formation. These conclusions underscore the potential of DNNs to enhance the understanding and prediction of protein functions, with implications for advancing therapies and diagnostics in regenerative medicine and dentistry.

## Figures and Tables

**Table 1:** Accuracy of the model.

Model	ACC	Sensitivity	Specificity	AUC	MCC
DNN	0.855	0.89	0.82	0.902	0.711

## Data Availability

The data that support the findings of this study are available upon request from the corresponding author.
